# The genome sequence of an ichneumonid wasp, *Campoletis raptor *(Zetterstedt, 1838)

**DOI:** 10.12688/wellcomeopenres.19738.1

**Published:** 2023-07-20

**Authors:** Gavin R. Broad, Chris Fletcher, Inez Januszczak, Natural History Museum Genome Acquisition Lab, Darwin Tree of Life Barcoding collective, Wellcome Sanger Institute Tree of Life programme, Wellcome Sanger Institute Scientific Operations: DNA Pipelines collective, Tree of Life Core Informatics collective, Darwin Tree of Life Consortium

**Affiliations:** 1 Natural History Museum, London, England, UK

**Keywords:** Campoletis raptor, an ichneumonid wasp, genome sequence, chromosomal, Hymenoptera

## Abstract

We present a genome assembly from an individual female *Campoletis raptor* (an ichneumonid wasp; Arthropoda; Insecta; Hymenoptera; Ichneumonidae). The genome sequence is 218.6 megabases in span. Most of the assembly is scaffolded into 11 chromosomal pseudomolecules. The mitochondrial genome has also been assembled and is 28.53 kilobases in length.

## Species taxonomy

Eukaryota; Metazoa; Eumetazoa; Bilateria; Protostomia; Ecdysozoa; Panarthropoda; Arthropoda; Mandibulata; Pancrustacea; Hexapoda; Insecta; Dicondylia; Pterygota; Neoptera; Endopterygota; Hymenoptera; Apocrita; Ichneumonoidea; Ichneumonidae; Campopleginae; Dusona group; *Campoletis*; *Campoletis raptor* (Zetterstedt, 1838) (NCBI:txid2922060).

## Background

*Campoletis raptor* is a small (5–8 mm body length), largely black ichneumonid wasp with the metasoma mostly red medially, and with the hind tibia banded black and pale red. The ovipositor is relatively long and apically strongly upcurved. As with most species of *Campoleti*s, the clypeus (below the face) has a median point. Campopleginae genera can be difficult to identify, although Klopfstein *et al.* (2022) have produced an interactive key to European genera. Riedel (2017) has revised the European species of *Campoletis*, with identification keys. The nominate subspecies of *C. raptor* ranges from Spain and the UK in the West to Bulgaria in the East, with a separate subspecies described for populations in Central Asia, in Kyrgyzstan and Turkmenistan ( Riedel, 2017). Very little is known about the distribution of *C. raptor* in Britain or Ireland, although there are museum specimens from England.

As with most species of the ichneumonid subfamily Campopleginae, *C. raptor* is a koinobiont endoparasitoid of Lepidoptera larvae. Oviposition is into a small larva which is killed in a later instar, with the wasp larva spinning a cocoon outside the host remains. A variety of lepidopteran hosts have been reported for *C. raptor*, but these are mostly dubious because of potential parasitoid misidentifications. Riedel (2017), in his recent taxonomic revision, only reported *Mythimna conigera* (Denis & Schiffermüller) (Brown-line Bright-eye) as a host, although it is likely other hosts are attacked too. *Campoletis* species often have distinctively patterned, black and white cocoons, presumably mimicking bird droppings to offer some defence as they pupate in relatively exposed positions, with the host larva killed before it is fully grown ( Broad *et al.*, 2018).

Although little is known of the biology of *C. raptor*, the North American *Campoletis sonorensis* (Cameron) has been intensively studied as it is an important natural enemy of an agricultural pest caterpillar, *Spodoptera frugiperda* (Smith) ( Isenhour, 1985; Isenhour, 1986). *Campoletis sonorensis* has a high fecundity and, as with other *Campoletis* species, pupation time in the cocoon can be rapid. Polydnaviruses are incorporated into the genome of *C. sonorensis*, helping the wasp larva overcome the host immune system ( Federici & Bigot, 2003). As more genomes of polydnavirus-carrying wasps are assembled, from congenerics to more distantly related subfamilies, we will gain greater understanding of the roles these viruses have played in the diversification of parasitoids.

## Genome sequence report

The genome was sequenced from one female *Campoletis raptor* collected from Wytham Woods, Oxfordshire (51.77, –1.31). A total of 83-fold coverage in Pacific Biosciences single-molecule HiFi long reads was generated. Primary assembly contigs were scaffolded with chromosome conformation Hi-C data. Manual assembly curation corrected 16 missing joins or mis-joins and removed 2 haplotypic duplications, reducing the assembly length by 0.97% and the scaffold number by 19.23%, and increasing the scaffold N50 by 2.46%.

**Figure 1.**
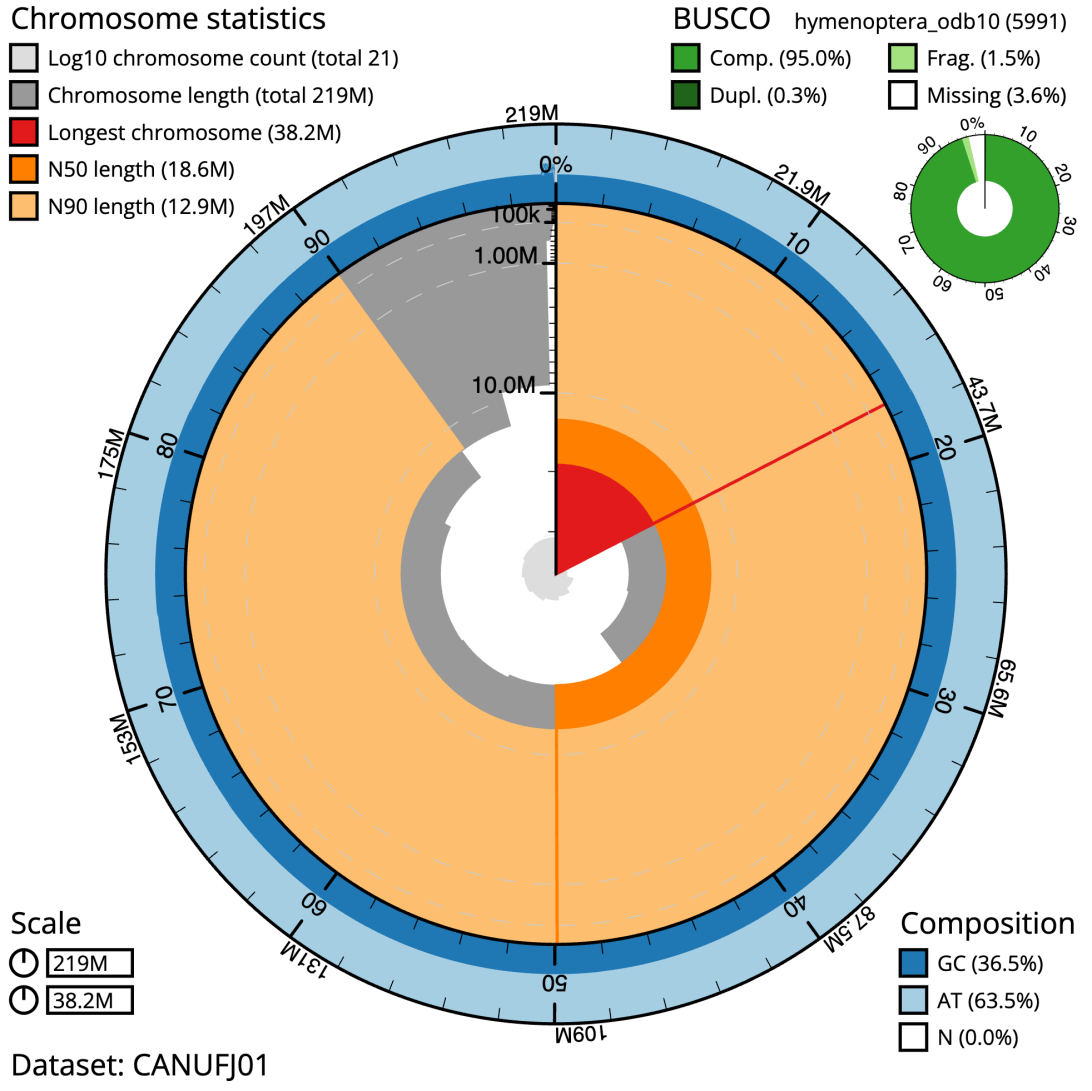
Genome assembly of *Campoletis raptor*, iyCamRapt1.1: metrics. The BlobToolKit Snailplot shows N50 metrics and BUSCO gene completeness. The main plot is divided into 1,000 size-ordered bins around the circumference with each bin representing 0.1% of the 218,627,199 bp assembly. The distribution of scaffold lengths is shown in dark grey with the plot radius scaled to the longest scaffold present in the assembly (38,199,786 bp, shown in red). Orange and pale-orange arcs show the N50 and N90 scaffold lengths (18,621,092 and 12,881,085 bp), respectively. The pale grey spiral shows the cumulative scaffold count on a log scale with white scale lines showing successive orders of magnitude. The blue and pale-blue area around the outside of the plot shows the distribution of GC, AT and N percentages in the same bins as the inner plot. A summary of complete, fragmented, duplicated and missing BUSCO genes in the hymenoptera_odb10 set is shown in the top right. An interactive version of this figure is available at https://blobtoolkit.genomehubs.org/view/iyCamRapt1.1/dataset/CANUFJ01/snail.

The final assembly has a total length of 218.6 Mb in 20 sequence scaffolds with a scaffold N50 of 18.6 Mb ( Table 1). Most (99.98%) of the assembly sequence was assigned to 11 chromosomal-level scaffolds. Chromosome-scale scaffolds confirmed by the Hi-C data are named in order of size ( Figure 1– Figure 4; Table 2). While not fully phased, the assembly deposited is of one haplotype. Contigs corresponding to the second haplotype have also been deposited. The mitochondrial genome was also assembled and can be found as a contig within the multifasta file of the genome submission.

**Table 1.** Genome data for *Campoletis raptor*, iyCamRapt1.1.

Project accession data		
Assembly identifier	iyCamRapt1.1	
Species	*Campoletis raptor*	
Specimen	iyCamRapt1	
NCBI taxonomy ID	2922060	
BioProject	PRJEB56062	
BioSample ID	SAMEA14448294	
Isolate information	iyCamRapt1, female: whole organism (DNA sequencing and Hi-C scaffolding)	
Assembly metrics *		*Benchmark*
Consensus quality (QV)	63.6	*≥ 50*
*k*-mer completeness	100%	*≥ 95%*
BUSCO **	C:95.0%[S:94.7%,D:0.3%], F:1.5%,M:3.6%,n:5,991	*C ≥ 95%*
Percentage of assembly mapped to chromosomes	99.98%	*≥ 95%*
Sex chromosomes	-	*localised homologous pairs*
Organelles	Mitochondrial genome assembled	*complete single alleles*
Raw data accessions		
PacificBiosciences SEQUEL II	ERR10224930	
Hi-C Illumina	ERR10297824	
Genome assembly		
Assembly accession	GCA_948107755.1	
*Accession of alternate haplotype*	GCA_948107775.1	
Span (Mb)	218.6	
Number of contigs	177	
Contig N50 length (Mb)	2.2	
Number of scaffolds	20	
Scaffold N50 length (Mb)	18.6	
Longest scaffold (Mb)	38.2	

* Assembly metric benchmarks are adapted from column VGP-2020 of “Table 1: Proposed standards and metrics for defining genome assembly quality” from ( Rhie *et al.*, 2021). ** BUSCO scores based on the hymenoptera_odb10 BUSCO set using v5.3.2. C = complete [S = single copy, D = duplicated], F = fragmented, M = missing, n = number of orthologues in comparison. A full set of BUSCO scores is available at https://blobtoolkit.genomehubs.org/view/iyCamRapt1.1/dataset/CANUFJ01/busco.

**Figure 2.**
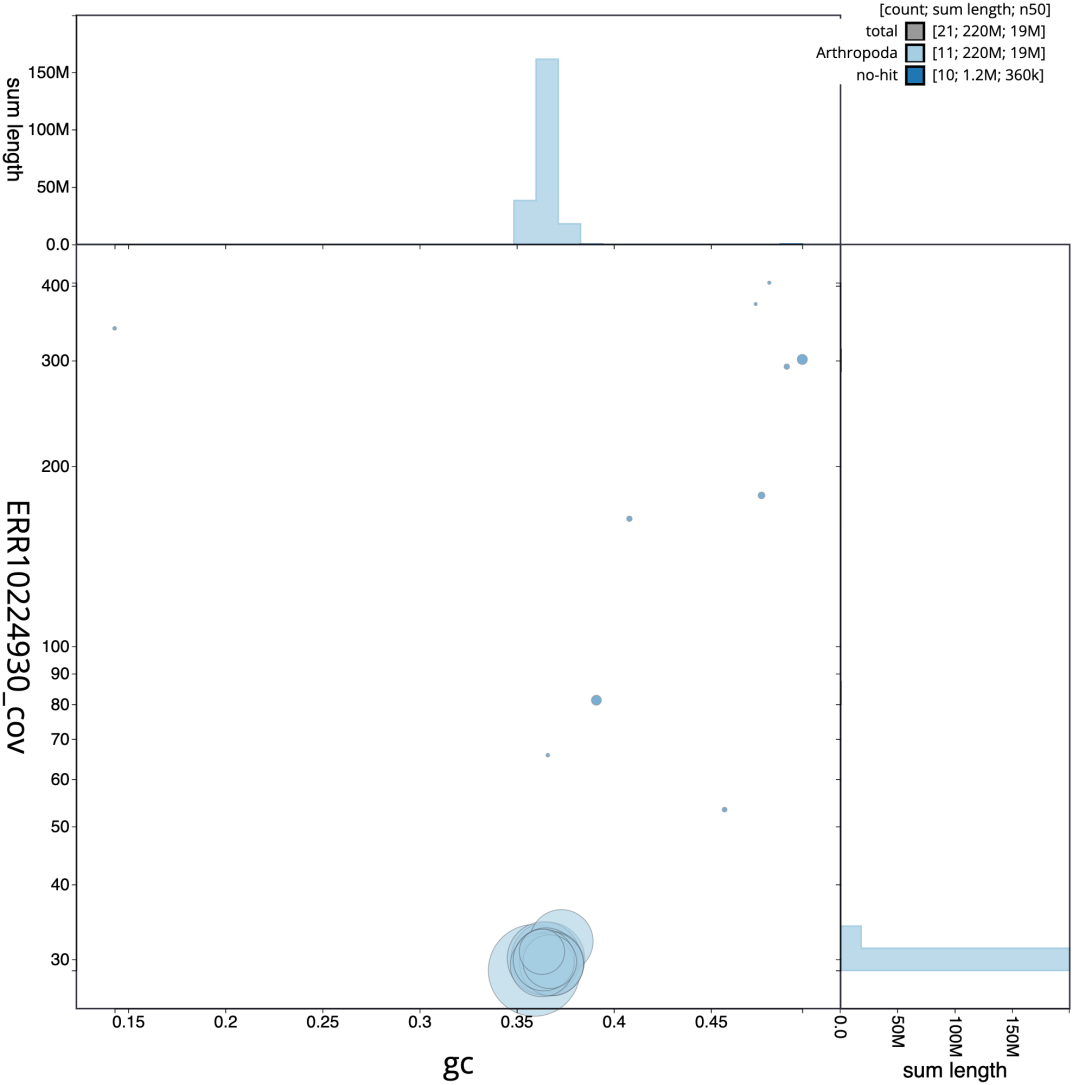
Genome assembly of *Campoletis raptor*, iyCamRapt1.1: BlobToolKit GC-coverage plot. Scaffolds are coloured by phylum. Circles are sized in proportion to scaffold length. Histograms show the distribution of scaffold length sum along each axis. An interactive version of this figure is available at https://blobtoolkit.genomehubs.org/view/iyCamRapt1.1/dataset/CANUFJ01/blob.

**Figure 3.**
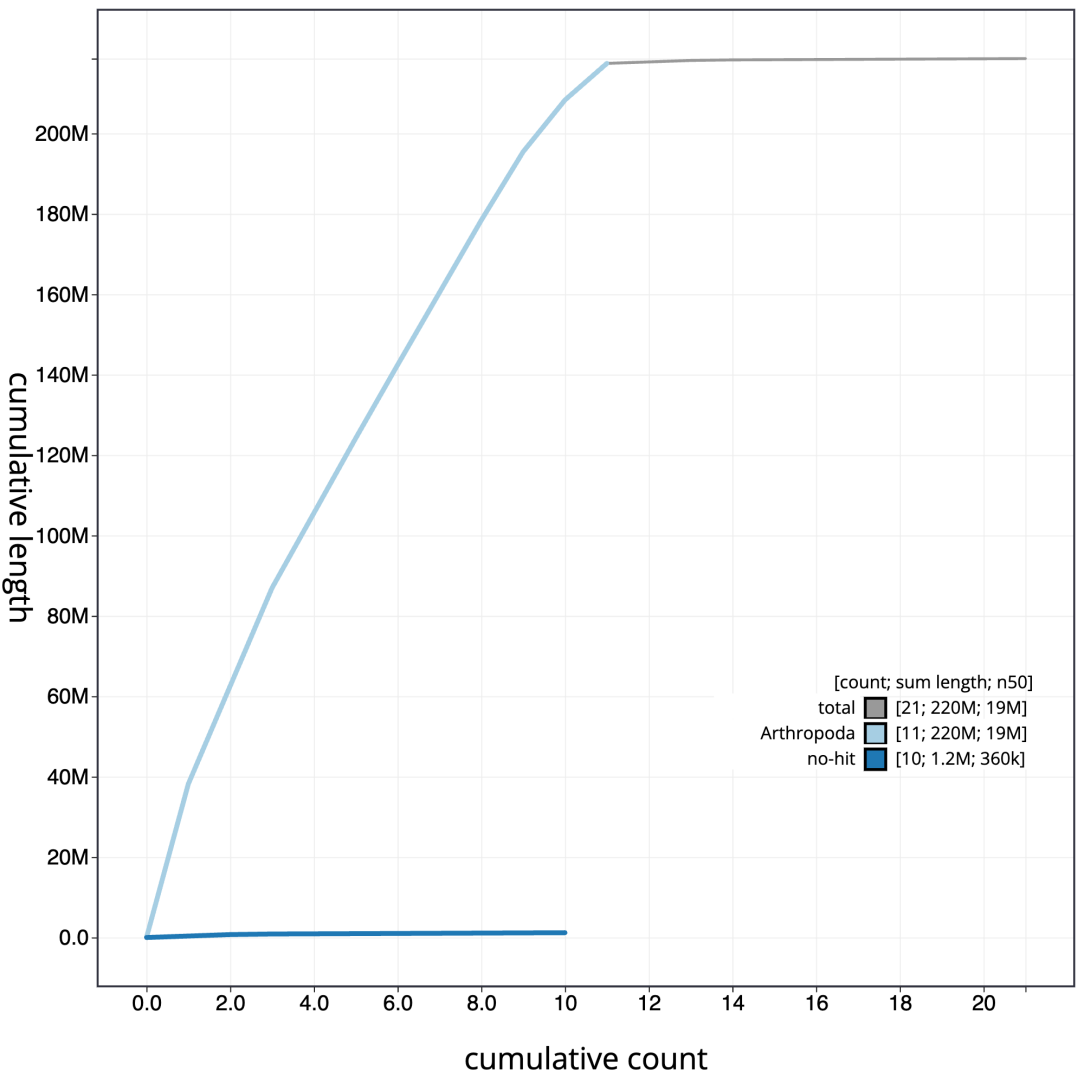
Genome assembly of *Campoletis raptor*, iyCamRapt1.1: BlobToolKit cumulative sequence plot. The grey line shows cumulative length for all scaffolds. Coloured lines show cumulative lengths of scaffolds assigned to each phylum using the buscogenes taxrule. An interactive version of this figure is available at https://blobtoolkit.genomehubs.org/view/iyCamRapt1.1/dataset/CANUFJ01/cumulative.

**Figure 4.**
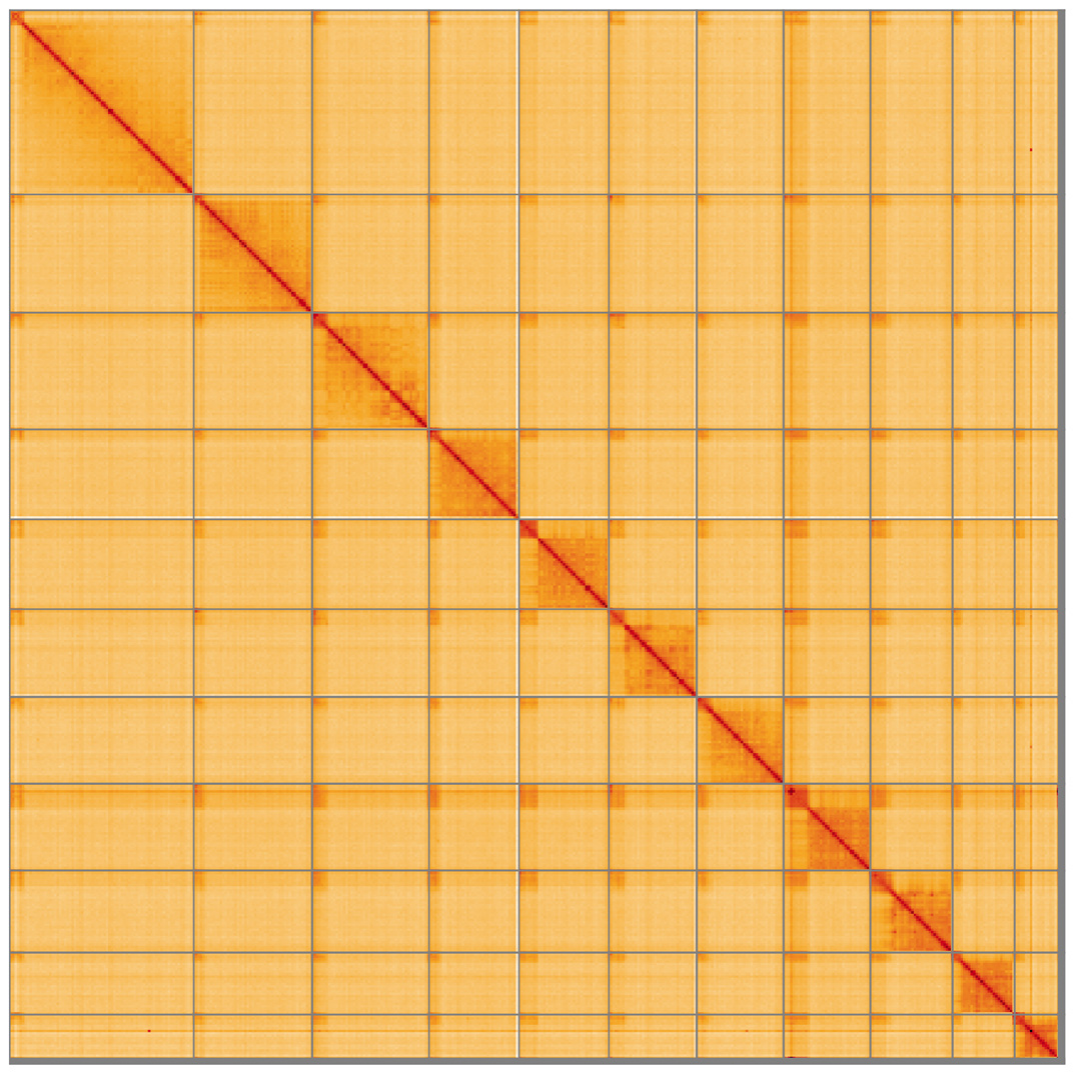
Genome assembly of *Campoletis raptor*, iyCamRapt1.1: Hi-C contact map of the iyCamRapt1.1 assembly, visualised using HiGlass. Chromosomes are shown in order of size from left to right and top to bottom. An interactive version of this figure may be viewed at https://genome-note-higlass.tol.sanger.ac.uk/l/?d=Enw_fapgTv2jUIWWQSOfRg.

**Table 2.** Chromosomal pseudomolecules in the genome assembly of *Campoletis raptor*, iyCamRapt1.

INSDC accession	Chromosome	Length (Mb)	GC%
OX403622.1	1	38.2	36.0
OX403623.1	2	24.52	36.5
OX403624.1	3	24.23	36.5
OX403626.1	5	18.65	36.5
OX403627.1	6	18.62	37.0
OX403628.1	7	18.21	37.0
OX403625.1	4	18.0	37.5
OX403629.1	8	18.0	36.5
OX403630.1	9	17.03	36.5
OX403631.1	10	12.88	36.5
OX403632.1	11	9.1	36.5
OX403633.1	MT	0.03	14.5

The estimated Quality Value (QV) of the final assembly is 63.6 with *k*-mer completeness of 100%, and the assembly has a BUSCO v5.3.2 completeness of 95.0% (single = 94.7%, duplicated = 0.3%), using the hymenoptera_odb10 reference set ( *n* = 5,991).

Metadata for specimens, spectral estimates, sequencing runs, contaminants and pre-curation assembly statistics can be found at https://links.tol.sanger.ac.uk/species/2922060.

## Methods

### Sample acquisition and nucleic acid extraction

A female *Campoletis raptor* (specimen ID NHMUK014451738, individual iyCamRapt1) was collected using an aerial net in Bert’s Pheasant Pen, Wytham Woods, Oxfordshire (biological vice-county Berkshire), UK (latitude 51.77, longitude –1.31) on 2021-09-02. The collectors were Gavin Broad, Chris Fletcher and Inez Januszczak (all Natural History Museum). The specimen was identified by Gavin Broad (Natural History Museum) and then dry frozen (–80°C).

The specimen was prepared for DNA extraction at the Tree of Life laboratory, Wellcome Sanger Institute (WSI). The iyCamRapt1 sample was weighed and dissected on dry ice with tissue set aside for Hi-C sequencing. Whole organism tissue was disrupted using a Nippi Powermasher fitted with a BioMasher pestle *.* DNA was extracted at the Wellcome Sanger Institute (WSI) Scientific Operations core using the Qiagen MagAttract HMW DNA kit, according to the manufacturer’s instructions.

### Sequencing

Pacific Biosciences HiFi circular consensus DNA sequencing libraries were constructed according to the manufacturers’ instructions. DNA sequencing was performed by the Scientific Operations core at the WSI on the Pacific Biosciences SEQUEL II (HiFi) instrument. Hi-C data were also generated from tissue of iyCamRapt1 using the Arima2 kit and sequenced on the Illumina NovaSeq 6000 instrument.

### Genome assembly, curation and evaluation

Assembly was carried out with Hifiasm ( Cheng *et al.*, 2021) and haplotypic duplication was identified and removed with purge_dups ( Guan *et al.*, 2020). The assembly was then scaffolded with Hi-C data ( Rao *et al.*, 2014) using YaHS. The assembly was checked for contamination and corrected as described previously ( Howe *et al.*, 2021). Manual curation was performed using HiGlass ( Kerpedjiev *et al.*, 2018) and Pretext ( Harry, 2022). The mitochondrial genome was assembled using MitoHiFi ( Uliano-Silva *et al.*, 2022), which runs MitoFinder ( Allio *et al.*, 2020) or MITOS ( Bernt *et al.*, 2013) and uses these annotations to select the final mitochondrial contig and to ensure the general quality of the sequence.

A Hi-C map for the final assembly was produced using bwa-mem2 ( Vasimuddin *et al.*, 2019) in the Cooler file format ( Abdennur & Mirny, 2020). To assess the assembly metrics, the *k*-mer completeness and QV consensus quality values were calculated in Merqury ( Rhie *et al.*, 2020). This work was done using Nextflow ( Di Tommaso *et al.*, 2017) DSL2 pipelines “sanger-tol/readmapping” ( Surana *et al.*, 2023a) and “sanger-tol/genomenote” ( Surana *et al.*, 2023b). The genome was analysed within the BlobToolKit environment ( Challis *et al.*, 2020) and BUSCO scores ( Manni *et al.*, 2021; Simão *et al.*, 2015) were calculated.

Table 3 contains a list of relevant software tool versions and sources.

**Table 3.** Software tools: versions and sources.

Software tool	Version	Source
BlobToolKit	4.1.7	https://github.com/blobtoolkit/ blobtoolkit
BUSCO	5.3.2	https://gitlab.com/ezlab/busco
Hifiasm	0.16.1-r375	https://github.com/chhylp123/ hifiasm
HiGlass	1.11.6	https://github.com/higlass/higlass
Merqury	MerquryFK	https://github.com/thegenemyers/ MERQURY.FK
MitoHiFi	2	https://github.com/marcelauliano/ MitoHiFi
PretextView	0.2	https://github.com/wtsi-hpag/ PretextView
purge_dups	1.2.3	https://github.com/dfguan/purge_ dups
sanger-tol/ genomenote	v1.0	https://github.com/sanger-tol/ genomenote
sanger-tol/ readmapping	1.1.0	https://github.com/sanger-tol/ readmapping/tree/1.1.0
YaHS	yahs- 1.1.91eebc2	https://github.com/c-zhou/yahs

### Wellcome Sanger Institute – Legal and Governance

The materials that have contributed to this genome note have been supplied by a Darwin Tree of Life Partner. The submission of materials by a Darwin Tree of Life Partner is subject to the **‘Darwin Tree of Life Project Sampling Code of Practice’**, which can be found in full on the Darwin Tree of Life website here. By agreeing with and signing up to the Sampling Code of Practice, the Darwin Tree of Life Partner agrees they will meet the legal and ethical requirements and standards set out within this document in respect of all samples acquired for, and supplied to, the Darwin Tree of Life Project. 

Further, the Wellcome Sanger Institute employs a process whereby due diligence is carried out proportionate to the nature of the materials themselves, and the circumstances under which they have been/are to be collected and provided for use. The purpose of this is to address and mitigate any potential legal and/or ethical implications of receipt and use of the materials as part of the research project, and to ensure that in doing so we align with best practice wherever possible. The overarching areas of consideration are:

•   Ethical review of provenance and sourcing of the material

•   Legality of collection, transfer and use (national and international) 

Each transfer of samples is further undertaken according to a Research Collaboration Agreement or Material Transfer Agreement entered into by the Darwin Tree of Life Partner, Genome Research Limited (operating as the Wellcome Sanger Institute), and in some circumstances other Darwin Tree of Life collaborators.

## Data Availability

European Nucleotide Archive: *Campoletis raptor.* Accession number PRJEB56062; https://identifiers.org/ena.embl/PRJEB56062. ( Wellcome Sanger Institute, 2023) The genome sequence is released openly for reuse. The *Campoletis raptor* genome sequencing initiative is part of the Darwin Tree of Life (DToL) project. All raw sequence data and the assembly have been deposited in INSDC databases. The genome will be annotated using available RNA-Seq data and presented through the Ensembl pipeline at the European Bioinformatics Institute. Raw data and assembly accession identifiers are reported in Table 1.

## Author information

Members of the Natural History Museum Genome Acquisition Lab are listed here: https://doi.org/10.5281/zenodo.4790042.

Members of the Darwin Tree of Life Barcoding collective are listed here: https://doi.org/10.5281/zenodo.4893703.

Members of the Wellcome Sanger Institute Tree of Life programme are listed here: https://doi.org/10.5281/zenodo.4783585.

Members of Wellcome Sanger Institute Scientific Operations: DNA Pipelines collective are listed here: https://doi.org/10.5281/zenodo.4790455.

Members of the Tree of Life Core Informatics collective are listed here: https://doi.org/10.5281/zenodo.5013541.

Members of the Darwin Tree of Life Consortium are listed here: https://doi.org/10.5281/zenodo.4783558.
