# Supplementary material for: The genome sequence of an ichneumonid wasp, Campoletis raptor (Zetterstedt, 1838)
Source: Wellcome Open Res. 2023 Jul 20;8:313. [Version 1] doi: 10.12688/wellcomeopenres.19738.1 (PMC11137483; doi:10.12688/wellcomeopenres.19738.1)
Supplement: Peer review history

# Reviewer report: Reviewer response for version 1

**DOI:** 10.21956/wellcomeopenres.21863.r83228

**Published:** 2024-05-29

**Authors:** Marko Prous

**Affiliations:** 1 Museum of Natural History, University of Tartu, Tartu, Estonia

The paper presents a genome sequence of *Campoletis raptor* (Ichneumonidae). The standard Darwin Tree of Life Consortium methods have been used. Seems that most or all the relevant papers have been cited to enable replication of the results more or less. Because of probably inevitable manual intervention during some  of steps, genome assembly is difficult to standardize in general. 

 I liked the background section providing main information about the organism (taxonomy, biology...), which is valuable addition to the genome report.

 In summary I don't have any specific criticism of the paper. My general suggestion would be to combine in the future at least some of the genome reports into one paper, combining based on taxonomic group or some other interesting aspect. Then there could be at least one additional aspect to that could make the paper more interesting and relevant. For example in case of Ichneumonidae or Ichneumonoidea a small comparative aspect of polydnaviruses in their genomes could be nice addition to standard genome reports. This could also make finding reviewers for the papers easier (fewer and more more interesting papers).

Are sufficient details of methods and materials provided to allow replication by others?

Partly

Is the rationale for creating the dataset(s) clearly described?

Yes

Are the datasets clearly presented in a useable and accessible format?

Yes

Are the protocols appropriate and is the work technically sound?

Yes

Reviewer Expertise:

Taxonomy, phylogeny, and genomics of sawflies (Hymenoptera).

I confirm that I have read this submission and believe that I have an appropriate level of expertise to confirm that it is of an acceptable scientific standard.

# Reviewer report: Reviewer response for version 1

**DOI:** 10.21956/wellcomeopenres.21863.r83233

**Published:** 2024-05-24

**Authors:** Željko Tomanović

**Affiliations:** 1 Serbia University of Belgrade, Belgrade, Serbia

The genome sequence of *Campoletis raptor* was presented by the authors. Although the biology of this species is not well known, the authors pointed out importance of the related North American *Campoletis sonorensis*, which is a natural enemy of *Spodoptera frugiperda*. The authors provided sufficient background information on these taxa and presented the genome of *C. raptor *in appropriate way.

Are sufficient details of methods and materials provided to allow replication by others?

Yes

Is the rationale for creating the dataset(s) clearly described?

Yes

Are the datasets clearly presented in a useable and accessible format?

Yes

Are the protocols appropriate and is the work technically sound?

Yes

Reviewer Expertise:

Taxonomy, Phylogenetics, biodiversity

I confirm that I have read this submission and believe that I have an appropriate level of expertise to confirm that it is of an acceptable scientific standard.
